# Single-cell analysis reveals XCL1+ CD8+ T cells as a therapeutic target in hepatocellular carcinoma

**DOI:** 10.1080/23723556.2025.2523085

**Published:** 2025-06-23

**Authors:** Guowei Li, Fan Yang, Zechao Li, Zhicheng Chen, Geng Luo, Hongtao Yuan, Chaoxian Zhao

**Affiliations:** a Department of Hepatobiliary Surgery, The First People’s Hospital of Guiyang, Guiyang, China; b Shanghai Cancer Institute, Renji Hospital Affiliated to Shanghai Jiao Tong University School of Medicine, Shanghai, China

**Keywords:** Immunotherapy, liver tumor, cell population

## Abstract

XCL1 (lymphotactin), a C-chemokine primarily produced by activated CD8+ T cells, remains poorly characterized in the context of immunotherapy. Here, we conducted comprehensive analyses based on multiple scRNA-seq datasets to identify the presence of XCL1+ CD8+ T cells in hepatocellular carcinoma (HCC) tumor microenvironment. Multiplex Immunohistochemistry and clinical data revealed that the infiltration of this cell population correlated with favorable outcomes. Cell–cell communication demonstrated interactions between XCL1+ CD8+ T cells and NK cells or myeloid cells via CD99 and MIF signaling pathways, respectively. These findings were further supported by spatial transcriptomic data. Using two independent bulk RNA-seq datasets, we found the mean of expression of XCL1 and CD8A could be an independent factor for prognosis of HCC, and next built a prediction score with five marker genes involved in XCL1+ CD8+ T cell population. Our findings proposed that XCL1 may play a key role in anti-tumor immunity and XCL1+ CD8+ T cell population could be a potential target to improve responses for immunotherapy in HCC.

## GRAPHICAL ABSTRACT

**Figure:**
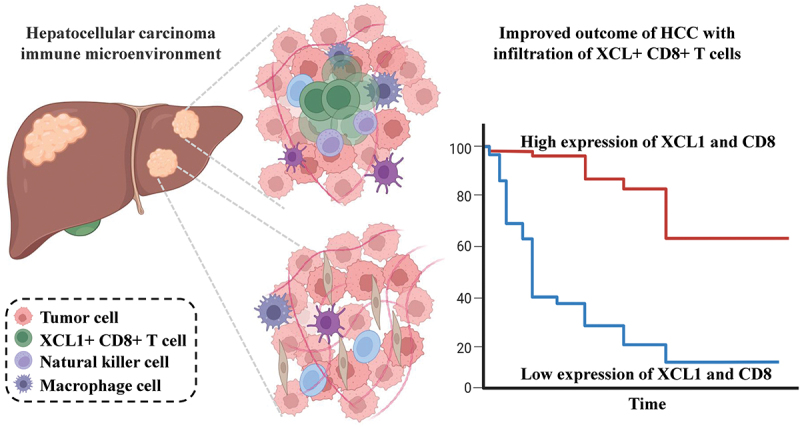

## Introduction

Based on GLOBOCAN data, liver cancer had ~865,000 new cases and 757,948 deaths in 2022, leading to liver cancer among the top three causes of cancer death worldwide. Its incidence and mortality rates are still rising.^1^ Hepatocellular carcinoma (HCC) accounted for about 85% to 90% of all primary liver cancers.^2^ Although alcohol-associated liver disease and diabetes mellitus are becoming more frequent risk factors, infection with hepatitis B or hepatitis C is still the major factor for the HCC development.^3–5^ Currently, there are multiple treatment options for HCC patients, such as curative resection, liver transplantation, chemoembolization, and immunotherapy.^5,6^ As one of immunotherapy, immune checkpoint inhibitors (ICIs) have shown the promising application of treatment, and revolutionized the management of HCC.^7^ However, it is well-known that only about 15~30% of patients have an effective response to immunotherapy. Moreover, recent studies showed that single-agent ICIs for advanced HCC only achieved the objective response rates of 15–20%, and most patients in clinical trials without a significant overall survival (OS) benefit.^5^

XCL1, a C class chemokine and also known as lymphotactin, is expressed in various immune cells mainly including CD8 T cells and NK cells.^8^ The chemotactic property of XCL1 is primarily for recruiting CD8 T cells to the tumor microenvironment to enhance the anti-tumor immune response.^9^ The detailed mechanism of recruitment is that XCL1 can interact with XCR1 predominantly expressed on dendritic cells (DCs),^10–12^ which is crucial for inducing tumor-specific CD8+ T cells. After recruitment, XCL1 further improves the activation and proliferation of CD8 T cells, and these CD8 T cells could produce various cytokines to increase cytotoxic activity against tumor cells. Emergency evidence suggests XCL1 is becoming a significant biomarker for multiple cancers including HCC,^13^ squamous cell carcinoma,^11^ and intestinal tumor,^14^ and targeting XCL1 could potentially induce anti-tumor immune responses.

In HCC, although there are only a few studies focused on its biological function, XCL1 shows promising application to improve the prognosis of HCC. The XCL1-Glypican-3 fusion gene has been developed as a cancer vaccine,^15^ showing promise in enhancing the generation of GPC3-specific CD8+ T cells, delaying liver cancer growth and improving the efficacy of anti-PD-1 therapy. This fusion protein also attracts dendritic cells, promoting a robust antitumor immune response. XCR1, receptor of XC1, is supposed to express in dendritic cell (DC). However, XCR1 expression was reported down-regulation in advanced HCC. Further experiments proved that overexpression of XCL1 inhibits HCC cell migration and invasion by affecting the epithelial–mesenchymal transition (EMT) and MAPK/PI3K/AKT signaling pathways.^16^ XCR1 is even suggested to be a prognostic marker that pinpoints targeted and immune-based therapy in hepatocellular carcinoma.^13^

In this study, we integrated spatial transcriptomic data, scRNA-seq, and bulk RNA-seq data to conduct system analyses to investigate the role of XCL1 in CD8+ T cells. We found that XCL1+ CD8+ T cells could communicate with NK and myeloid cells through CD99 and MIF signaling pathways to regulate the anti-tumor immunity of CD8+ T cell in HCC TME. Survival analysis showed that the mean of XCL1 and CD8A expression can be an independent prognostic factor for HCC, and the prediction score based on five marker genes had an efficient prediction for prognosis of HCC. Our analyses revealed the transcriptional characteristics of XCL1+ CD8+ T cells in HCC TME and suggested that this population is an important candidate targets for the therapy to improve outcomes of HCC.

## Methods

### Datasets

All scRNA-seq (n = 3) and bulk RNA-seq (n = 2) datasets used in this study were public and collected from high-quality publications. The Cell Ranger outputs for three scRNA-seq datasets were downloaded from the Gene Expression Omnibus (GSE149614, GSE156625, and GSE189903),^17–19^ respectively. For two bulk RNA-seq data, the Cancer Genome Atlas Liver Hepatocellular Carcinoma (TCGA-LIHC) cohort was obtained from GDC Data Portal (https://portal.gdc.cancer.gov/).^20^ The raw count files were generated by HTSeq, and we then applied TMM (trimmed mean of *M* values) normalization to raw count matrix for the following analysis. Additionally, the last quantitated gene expression matrix (called by Gao cohort) came from RSME software with upper quartile normalization was downloaded from NODE (accession code: OEP000321) (https://www.biosino.org/node/).^21^ The log2-transformed was further conducted for the matrix of Gao cohort.

### Pre-processing for scRNA-seq data

We used the Python package Scanpy to conduct the quality control and preprocessing of three scRNA-seq data independently.^22^ Cells with detected genes less than 200 and genes with detection in less than three cells were first excluded. We then filtered out cells with a percentage of expressed mitochondrial genes higher than 20%. Finally, the samples with high-quality cells less than 500 were excluded for further analysis.

### Integration, clustering, and annotation of scRNA-seq data

To remove the batch effects between different patients, the Python package scVI,^23^ using probabilistic modeling analysis of single-cell omics data, was used to integrate scRNA-seq data from 51 hCC samples. The scVI integration was performed by model training with the count matrix in raw object and then estimated the latent representation of each cell. Furthermore, “scanpy.pp.neighbors”, “scanpy.tl.umap”, and “scanpy.tl.leiden” function in Scanpy were used to conduct cell cluster analysis. The sub-clustering for CD8+ T cells had been performed by a similar pipeline.

To identify the differential gene expression for each cluster, we first normalized the raw counts using the function “normalize_total” and then transformed the data using the function “log1p”. The Wilcoxon rank-sum test in Scanpy (scanpy.tl.rank_genes_groups) was used to identify differential expression genes determined by log(fold change) > 0.25 and adjusted P-value < .05. Finally, the cell populations were annotated by combining the result of differential gene expression analysis for each cluster and the known marker genes.

### Trajectory inference for CD8+ T cells

The partition-based graph abstraction (PAGA) method in Scanpy was applied for trajectory analysis of CD8+ T cells in HCC.^24^ The PAGA graph was generated by the Leiden algorithm and visualized by denoising the graph. Finally, the marker genes for SELL+ CD8+ T cells, XCL1+ CD8+ T cells, and GZMA+ CD8+ T cells were used to construct gene changes along PAGA paths. All reconstructions of trajectory in this study were conducted with the default parameters.

### Cell communication analysis

The CellChatDB, including a comprehensive signaling ligand–receptor interactions, such as multimeric ligand–receptor complexes and stimulatory and inhibitory membrane-bound coreceptors, was used in this study. Cell–cell interaction analysis for HCC scRNA-seq data was conducted by the CellChat package.^25^ Based on the calculation of ensemble average expression of differentially expressed signaling genes, the CellChat quantifies the intercellular communication probability to explore the significant differences in ligand and receptor interactions between two cell clusters. Next, we performed systems analysis of cell–cell communication network to identify signaling roles (dominant senders, receivers, mediator, and influencer) of XCL1+ CD8+ T cells as well as the major contributing signaling. Finally, we generated scatter plot to visualize the dominant senders (sources) and receivers (targets) of each CD8+ subpopulation T cells in a 2D space.

### Spatial transcriptomic data analysis

The scanpy package was used to analyze the spatial transcriptomics data for HCC. We first imported the downloaded Visium data through read_visium() function. To avoid the bias from low-quality spots, we filtered spots based on total counts and expressed genes. After quality control and preprocessing, we conducted the normalization and clustering. Finally, the scanpy.pl.spatial() function was applied to overlay spots on top of the hematoxylin and eosin stain (H&E) image.

### Survival analysis

To determine the optimal cut-point for the gene expression value or risk score, we applied surv_cutpoint function in R package survminer (https://CRAN.R-project.org/package=survminer) to calculate a value that correspond to the most significant relation with the outcome of HCC patients in bulk RNA-seq cohort, respectively. The Kaplan–Meier method with a log-rank test was used to generate survival curves (https://CRAN.R-project.org/package=survival). The significance threshold (*p* value) in the survival analysis was ≤0.05. Hazard ratio (HR) between two groups was estimated by the Cox regression model. To determine whether the mean of gene expression of XCL1 and CD8A was an independent prognostic factor, the multivariate Cox regression analysis was performed in survival analysis.

### Multiplex immunohistochemistry (mIHC) staining

The mIHC was performed following the standard protocol by AlphaXPainter X30 (Alpha X Bio, Beijing, China) with 3-plex-4-color panel. The primary antibodies in the 3-plex-4-color panel were CD3E (Proteintech 60,181‐1‐Ig), CD8A (Proteintech 66,868‐1‐Ig), and XCL1 (Proteintech 26,605–1-AP), respectively. For the visualization, each stained slide was scanned by ZEISS AXIOSCAN 7 (ZEISS, Oberkochen, Germany).

## Results

### The high expression of XCL1+ CD8+ T cells in TME related to better outcomes of HCC

**Figure 1.**
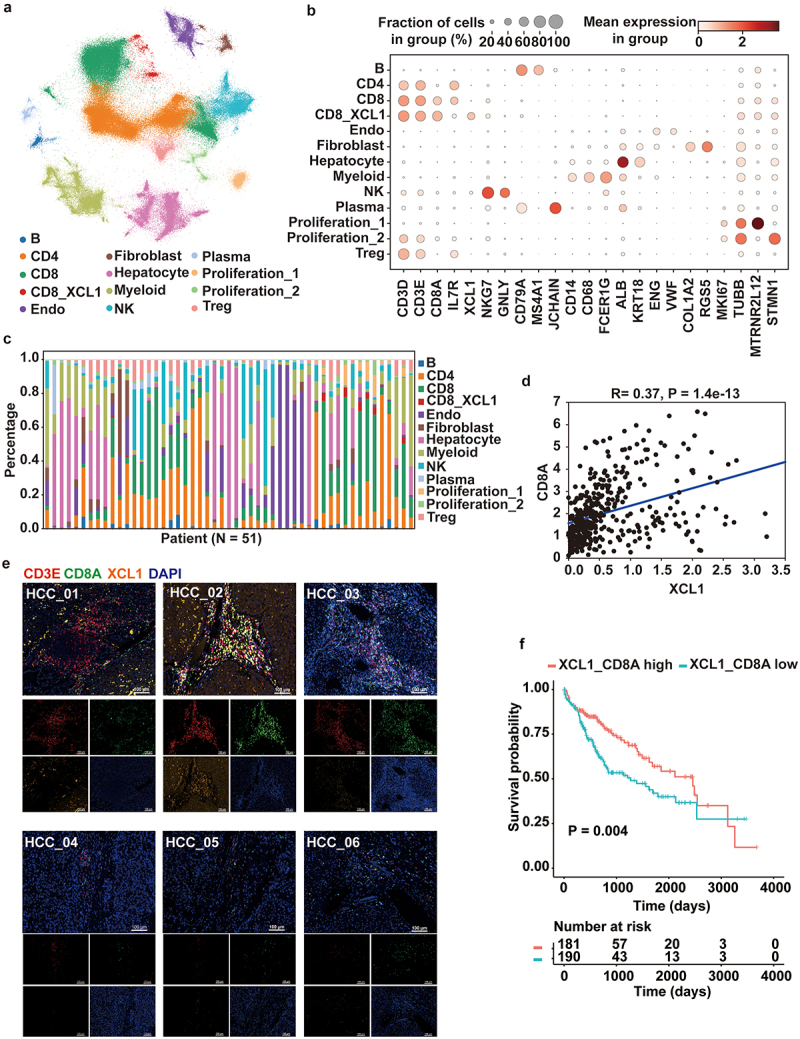
The presence of XCL1+ CD8+ T cells positively related to better outcomes in hepatocellular carcinoma (HCC). (a) Uniform manifold approximation and projection (UMAP) plot of integrated single-cell RNA-sequencing (scRNA-seq) data from 185, 658 high-quality cells obtained from HCC 51 samples. Cell clusters found therein representing 13 cell types are shown. (b) Dot plot showing expression levels of specific markers in each cell type. (c) Stacked plot showing the proportion of cells that contributed to each cell type by each sample. (d) *XCL1* expression positively correlated to the expression of *CD8A* in HCC from TCGA cohort. (e) Multiplex immunohistochemistry (mIHC) staining of three markers (*CD3E*, *CD8A* and *XCL1*) in three patients with good (upper panel) and bad (lower panel) outcomes. Each marker was represented by a different color, as indicated in the panel. Scale bars: 100 µm. (f) Kaplan–Meier survival analysis for two groups defined by patients in the cancer genome atlas (TCGA)-LIHC cohort with high/low mean of expression *XCL1* and *CD8A*. The sample numbers for each group are shown in brackets. Statistical significance is determined by log-rank test.

To explore the cell type diversity in HCC at single-cell resolution, we used scRNA-seq to examine liver tissues collected from 51 samples with tumor, adjacent-tumor, and healthy. The number of cells per sample was higher than 500 cells, and most of the samples included more than 1000 cells to generate detailed atlas of cell states and transcriptional programs in the TME of HCC. After quality control removing cells with less than 200 genes expressed and genes that are detected in less than 3 cells, we totally obtained 185, 658 high-quality cells for further analysis. We finally used the scVI method to perform the integration of three independent scRNA-seq cohorts (Figure 1a,b).

In this study, we found that there are three unconventional cell populations including two proliferation populations with high expression of Ki67 gene and one specific CD8+ T cells with extremely high expression of XCL1 gene (Figure 1b-d). In addition, the TME in HCC mainly includes hepatocytes (ALB+ and KRT18+), endothelial cell (ENG+ and VWF+), B cells (CD79A+ and MS4A1+), Plasma (CD79A+ and JCHAIN+), T cells (CD3D+ and CD3E+), myeloid (CD68+ and FCER1G+), NK cells (NKG7+ and GNLY+), and few fibroblasts (COL1A2+ and RGS5+). Consistent with previous results, the composition of cell types in HCC TME has been significantly heterogeneous in various patients (Figure 1c).

To validate the expression of XCL1 in CD8+ T cells and investigate the potential role of this population in HCC, we first analyzed bulk RNA-seq data from TCGA-LIHC cohort. After normalization of gene expressions, we first calculated the correlation analysis of gene expression between CD8 and XCL1. Results showed that XCL1 expression was positively associated with CD8 expression in the TCGA cohort (*R* = 0.37, *p* = 1.4e-13) (Figure 1d). We further conducted multiplex immunohistochemistry (mIHC) technology to validate the presence of XCL1+ CD8+ T cells in HCC. The results showed the infiltration of XCL1+ CD8+ T cells in three HCC patients with better outcomes (overall survival >5 y) compared to the other three HCC patients with poorer outcomes (Figure 1e). Combined with the clinical data, we also conducted survival analysis to examine the potential associations between the expression of XCL1 and CD8 and survival of patients in TCGA-LIHC cohort, suggesting that high mean of expression of XCL1 and CD8 (*n* = 181, TPM value > 1.224) is positively correlated with better prognosis of HCC.

### Subpopulation analysis of CD8+ T cells and trajectory inference for XCL1+ CD8+ T cells

**Figure 2.**
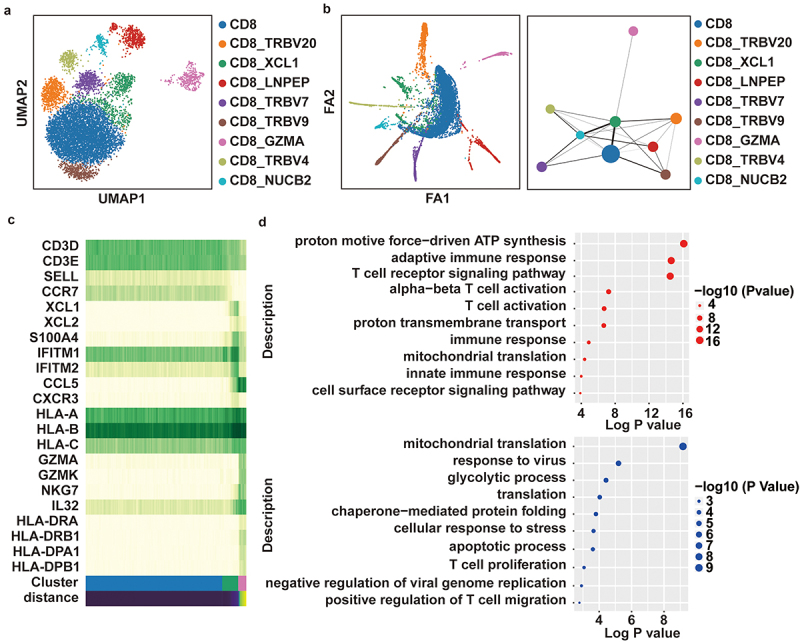
Trajectory inference for XCL1+ CD8+ T cells. (a) uniform manifold approximation and projection (UMAP) plot of nine subpopulations of CD8+ T cells marked by *SELL*, *TRBV20, XCL1*, *LNPEP*, *TRBV7*, *TRBV9*, *GZMA*, *TRBV4* and *NUCB2*, respectively. (b) The partition-based graph abstraction (PAGA) embedding plot showing the trajectory inference of CD8+ T subpopulations. The width of line representing the connectivity adjacency between clusters. Dot plot showing expression levels of specific markers in each cell type. (c) The dynamic expression pattern of marker genes (*SELL*, *XCL1*, *IFITM1*, *GZMA*, HLA-I, and HLA-II genes) along PAGA path. (d) Gene ontology pathway enrichment analysis of differential gene expressions (log2 Fold change ≥ 0.25 or ≤ 0.25 and false discovery rate ≤ 0.05) between XCL1+ CD8+ T cells and the rest of all cells including hepatocyte, B, NK, fibroblast cell etc. (upper panel), between XCL1+ CD8+ T cells and other CD8+ T cells (lower panel).

To investigate the transcriptional characteristics of XCL1+ CD8+ T cells and their differentiation trajectory in HCC, we used sample (called by S3T2) from GSE189903 cohort with 8,493 CD8+ T cells including 609 XCL+ CD8+ T cells to perform subpopulation analysis and trajectory inference. In S3T2 hCC, we found that there were at least nine subpopulations of CD8+ T cells. The largest proportion of population was high expression of SELL and CCR7 gene. Exception part populations with high expression of various human T cell receptor beta variable (TRBV) genes, such as TRBV20, TRBV7, TRBV9, and TRBV4, we found two specific populations of XCL1+ CD8+ T cell and GZMA+ CD8+ T cells (Figure 2a). Furthermore, trajectory inference revealed that XCL1+ CD8+ T cells probably have strong development ability to other CD8+ T subpopulations, especially for GZMA+ CD8+ T cells (Figure 2b), suggesting that probably plays a key role in anti-tumor immunity.

Next, we used marker genes for SELL+ CD8+ T cell, XCL1+ CD8+ T cell, and GZMA+ CD8+ T cell to reconstruct gene changes along PAGA paths. We found that XCL2, S100A4, IFITM1, IFITM2, and HLA-I type genes were significantly high expression in XCL1+ CD8+ T cells (Figure 2c). XCL2 is supposed to be another chemokine (C motif) ligand in the C-chemokine family. Notably, interferon-induced transmembrane protein 1 (IFITM-1) and IFITM-2 have been reported to exhibit a broad spectrum of antiviral activity, specifically as a potent antiviral effector against hepatitis C virus (HCV). The Gene ontology based two datasets of different expression genes (XCL1+ CD8+ T cell vs all other cells (including hepatocytes, B cell, NK, etc.); XCL1+ CD8+ T cell vs other CD8+ T cells), also supported that XCL1+ CD8+ T cells play a key role in anti-tumor immunity, specifically in HCC patients with virus infections (Figure 2d).

### Cell–cell communication revealing the XCL1+ CD8+ T cell cooperation with NK and myeloid cells

**Figure 3.**
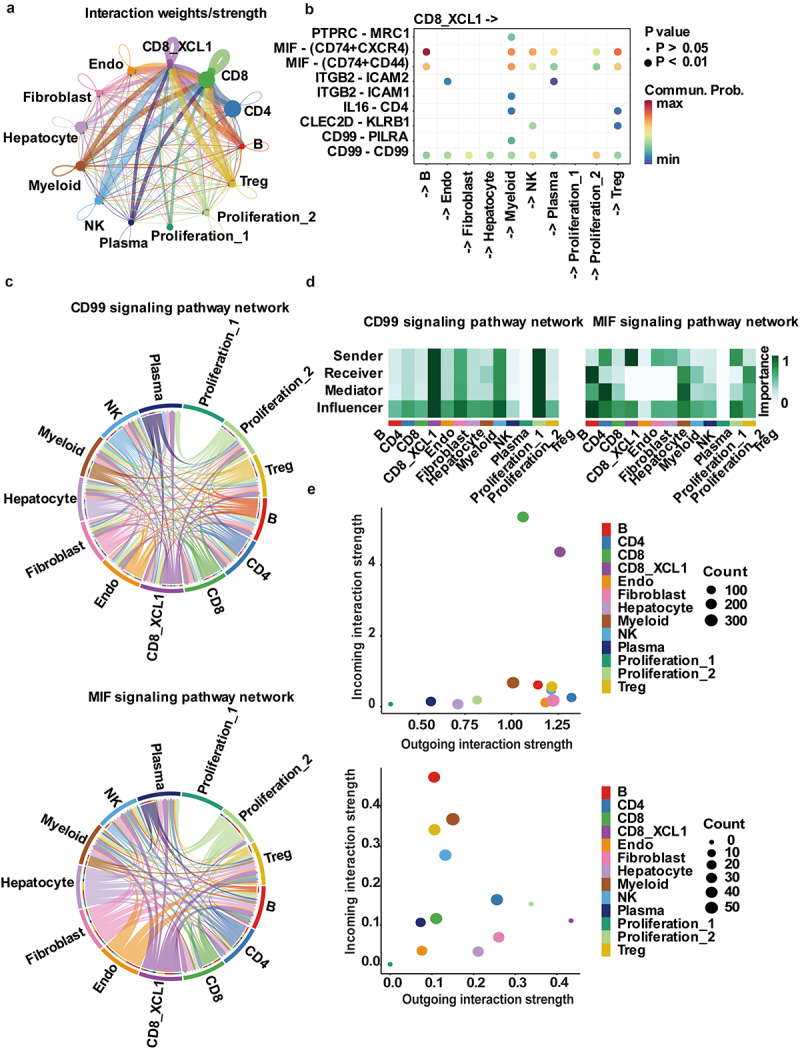
Cell–cell communication of XCL1+ CD8+ T cells in HCC TME. (a) Circle plots showing putative ligand–receptor interactions between XCL1+ CD8+ T cells and other cell clusters, with the width of edges representing the strength of the communication. (b) The heatmap showing the significant interaction between XCL1+ CD8+ T cells and other populations mainly through CD99 and MIF signaling pathways. Each row represents a ligand–receptor pair and each column defines a cell–cell interaction pair. (c) Chord diagram plot for the aggregated cell–cell communication network at CD99 signaling pathway (upper panel), and MIF signaling pathway (lower panel), respectively. (d) Heatmap plot for the centrality scores of CD99 signaling pathway network (left panel), and MIF signaling pathway network (right panel). (e) Scatter plot showing the XCL1+ CD8+ T cell population both as dominant senders (sources/outcoming interaction) and receivers (targets/incoming interaction) in CD99 signaling pathway (upper panel), and the dominant senders (sources/outgoing interaction) only in MIF signaling pathways (lower panel).

To explore the complex network of communication involved in XCL1+ CD8+ T cells, we identified putative cell–cell interactions by CellChat. As expected, interactions involving XCL1+ CD8+ T cells and other immune cells dominated the TME networks (Figure 3a). The ligand–receptor interactions in HCC TME were mainly through CD99 and MIF signaling pathways (Figure 3b,c). The XCL1+ CD8+ T cells had strong intercellular interactions with NK and myeloid cells mainly through CD74 and CXCR4, CD74 and CD44, and CD99 and CD99 axis, respectively (Figure 3B).

To investigate the signaling roles of XCL1+ CD8+ T cells in the intercellular communication network, we used measures in weighted-directed networks to identify the dominant cell senders and receivers of signaling networks. Our results suggested that XCL1+ CD8+ T cells play the complicated role in CD99 signaling pathway network, as both sender and receiver (Figure 3d). At the same time, XCL1+ CD8+ T cells are only activated as a sender in MIF signaling pathway (Figure 3d). The scatter plot in a 2D space also showed that the strength of XCL1+ CD8+ T cells is both high in incoming and outgoing interaction in CD99 signaling, while the incoming interaction strength (receiver) of XCL1+ CD8+ T cells is low in MIF signaling (Figure 3e). Due the CD99 and MIF signaling pathways both having a prominent function in cell adhesion, migration, survival and death, the therapy targeting for XCL1+ CD8+ T cells might be an efficient treatment to improve prognosis of HCC.

### Collocating XCL1+ CD8+ T cells with NK and myeloid cells validated by spatial transcriptome data

**Figure 4.**
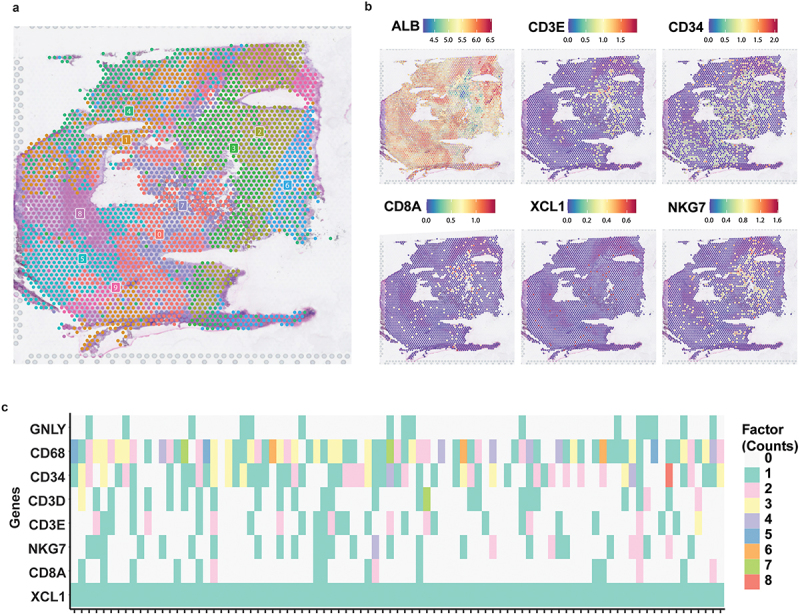
Spatial transcriptome data supporting the co-location of XCL1+ CD8+ T cells with NK and myeloid cells. (a) a pathologic section from tumor tissues of one HCC patient (S3T2). Annotations obtained by cluster analysis of the HCC. (b) The expression level of *ALB*, *CD3E*, *CD34*, *CD8A*, *XCL1*, and *NKG7* in S3T2 spots. (c) Heatmap showing the 91 spots all expressed *XCL1* in region of cluster2 and cluster3, and most of these spots also expressed immune cell marker genes (*CD8A*, *NKG7*, *CD3E*, *CD3D*, *CD34*, *CD68*, and *GNLY*). Different color means the number of reads of genes in a spot.

To examine spatially transcriptional characteristics of XCL1+ CD8+ T cells within liver tissues, we performed the unsupervised leiden-clustering to unravel the spatial location in cell expression profiles (Figure 4a). Given the low expression of ALB and high expression of immune cell markers of CD3E and CD34, we annotated the region of cluster2 and cluster3 as the tissue with enrichment of immune cells based on the spatial expression pattern. We further investigated the spatial expression of CD8A, XCL1, and NKG7 in a spatial transcriptomics slice, suggesting that all of three markers were comparatively highly expressed in the region of cluster2 and cluster3 (Figure 4a,b). Next, we found that there were ~90 spots with expression of XCL1 in the region of cluster2 and cluster3, and then counted the number of reads of main immune cell marker genes among these spots, suggesting the co-expression of XCL1, CD8A, NKG7, CD68, and CD34 in cluster2 and cluster3 (Figure 4c). Taken together, our spatial transcriptomics analysis supported that the XCL1+ CD8+ T cells could communicate with NK and myeloid cells to play an anti-tumor immunity role in HCC.

### Constructing prediction score at bulk RNA-seq data

**Figure 5.**
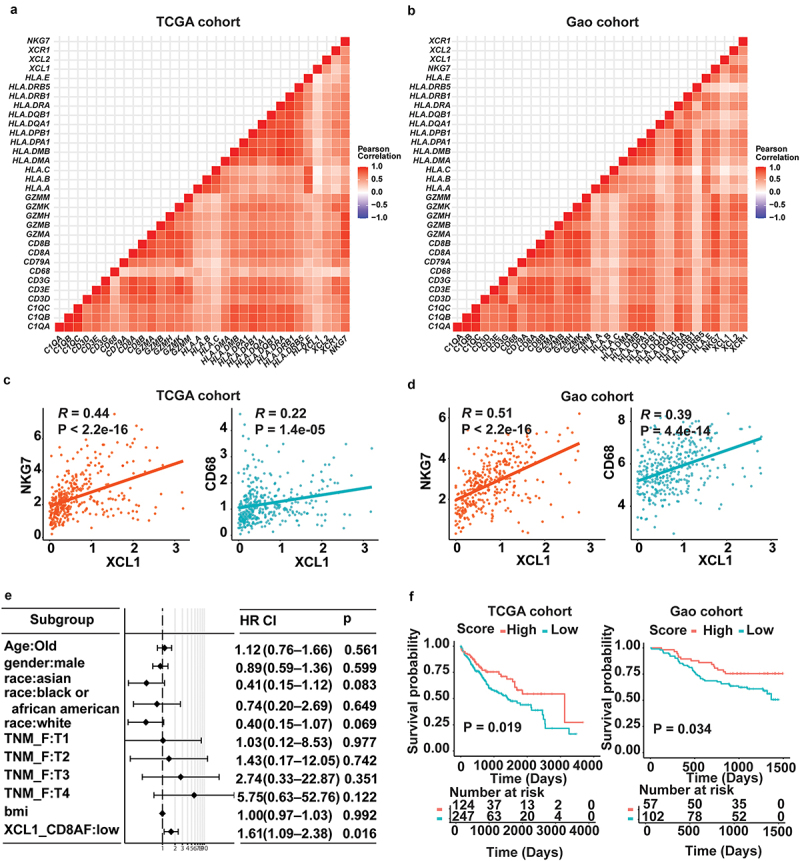
Survival analysis showing high expression of XCL1+ CD8+ T cells as independent prognosis factor for HCC. (a) Significantly positive association between *XCL1* and other immune cell signature genes (such as T cell, B cell, and myeloid cells) in TCGA cohort. The correlation coefficients were measured by Pearson correlation. (b) Consistent association found in Gao cohort. (c) Significantly positive between *XCL1* and NK signature genes *NKG7* association (*R* = 0.44, *p* < 2.2e-16), and between *XCL1* and myeloid signature gene *CD68* (*R* = 0.22, *p* = 1.4e-05) in TCGA. The correlation coefficients were measured by Pearson correlation. (d) Validation of positive association by Gao cohort (*NKG7*, *R* = 0.51, *p* < 2.2e-16; *CD68*, *R* = 0.39, *p* = 4.4e-14, respectively). (e) Multivariate analysis showing the mean of expression of *XCL1* and *CD8A* was an independent risk factor for survival of HCC. (f) Kaplan–Meier survival analysis for two groups defined by patients in the cancer genome atlas (TCGA)-LIHC cohort and Gao cohort with high/low mean expression of *XCL1* and *CD8A*, respectively. The sample numbers for each group are shown in brackets. Statistical significance is determined by log-rank test.

As the above analyses performed at single-cell, we next validated the positive correlation between XCL1+ CD8+ T cells and NK cell, and XCL1+ CD8+ T cells and myeloid cells using two independent bulk RNA-seq datasets with a total of more than 500 hCC patients (Figure 5a,b). It should be mentioned that the expression of XCL1 in HCC was comparatively low and bulk RNA-seq data was mixed by diverse cell types. However, our correlation analyses were consistent with the results from scRNA-seq data, suggesting the valid correlation between XCL1 and NKG7 (TCGA cohort, *R* = 0.44, *p* < 2.2e-16; Gao cohort, *R* = 0.51, *p* < 2.2e-16), XCL1 and CD68 (TCGA cohort, *R* = 0.22, *p* = 1.4e-05; Gao cohort, *R* = 0.39, *p* < 4.4e-14) (Figure 5c,d), respectively.

To test whether the mean of expression of XCL1 and CD8A can be an independent predictor for HCC prognosis, we conducted multivariable analysis to show that the low expression of XCL1 and CD8A is the independent prognostic factor for worse outcomes of HCC (*p* = .016, HR = 1.61) (Figure 5e). For other risk factors, our results revealed that the race and TNM staging tended to be the independent factors for HCC, consistent with the previous studies. Finally, we counted the mean expression of XCL1, CD8A, NKG7, XCL2, and XCR1 as prediction score to classify HCC patients. Our survival results showed that this prediction score with five genes can efficiently predict the prognosis of HCC in two independent cohorts (TCGA cohort, *p* = .019; Gao cohort, *p* = .034) (Figure 5f).

## Discussion

As a C class chemokine, XCL1 has been found to play a key role in anti-tumor immune response in the recent years.^12^ Here, our findings were consistent with previous studies, suggesting that the expression of XCL1 has been positively related to CD8+ T cells and NK cells. Moreover, we integrated efficient scRNA-seq and spatial transcriptome data to reveal the characteristics of this new subpopulation of XCL1+ CD8+ T cells at single-cell levels. We found the significant cell–cell communication between XCL1+ CD8+ T cells and NK cells through CD99 and MIF signaling pathways and XCL1 + CD8+ T cells and myeloid cells through MIF signaling pathway. Spatial data further supported the collocations between these cell populations. Finally, we developed the XCL1 score including five genes related to XCL1+ CD8+ T cell population to efficiently screen the HCC patients with better prognosis. Taking together, our study showed that XCL1+ CD8+ T cell population is an important target for immunotherapy to improve outcomes of HCC.

The main problem for playing the anti-tumor role of XCL1+ CD8+ T cells is the low expression of XCL1 and key related genes (XCR1 and XCL2) in tumor microenvironment of HCC. In this study, the expression XCL1, XCR1 and XCL2 were comparatively low both in scRNA-seq data and bulk RNA-seq. We even have not identified the expression of XCR1 at part of patients. Due to the interaction between XCL1 in CD8+ T cells and XCR1 in DCs playing the key role in anti-tumor efficacy,^26,27^ the number of XCL1+ CD8+ T cells and XCR1+ DCs in microenvironment directly determined the prognosis of HCC patients. In the previous studies, the vaccine had been developed to increase the expression of XCL1+ CD8+ T cells or XCR1+ DCs, separately, leading to induce potent anti-tumor immunity and kill the tumor cells in few tumors, especially in HCC and lung cancer.^28,29^ In addition, the overexpression of XCR1 was correlated to better outcomes in HCC patients with the treatment of sorafenib.^13^ Co-activation of XCL1 and XCR1 probably enhances the high anti-tumor efficacy and improves the prognosis of HCC.

We found that the pathway involved mitochondrial function, especially the mitochondrial translation and glycolytic process, had been enriched in XCL1+ CD8+ T cells compared to other cell populations. Due to the immune cells playing a key role in drug resistance and anti-tumor function,^30^ and the necessary of mitochondria for establishing immune cell phenotype and their function,^31,32^ our results suggested that regulating mitochondria probably is one of useful strategy to increase the infiltration of XCL1+ CD8+ T cells in tumor.

Integration of cell–cell communication and spatial transcriptome analysis also showed that XCL1 probably is a key factor in regulating the immune microenvironment of HCC. During the inflammatory and immunological responses, XCL1 usually has been activated to involve in cross-presentation, antigen uptake, and induction of innate as well as adaptive cytotoxic immunity. Given the key role of XLC1 in tumor immunity microenvironment, the XCL1 becomes an important factor to regulate anti-tumor immunity. Our cell–cell communication analysis suggested that XCL1+ CD8+ T cells can cooperate with NK cells and myeloid cells (probably DCs), but also other immune cells, such as CD4+ T cells and B cells. Spatial data further supported the co-location of these immune subpopulations. In future work, the system study of function of XCL1 in anti-tumor immunity would improve the outcomes of tumor patients.

In conclusion, our findings highlighted the presence of XCL1+ CD8+ T cells in HCC TME and then characterized the gene expression signature of this population in HCC and observed dramatically elevated XCL1 expression in CD8+ T cells positively related to better outcomes. Integration analysis of spatial transcriptome, scRNA-seq, and bulk RNA-seq data further revealed that the potential interactions between XCL1+ CD8+ T cells and NK cells, and XCL1+ CD8+ T cells and myeloid cells, play the key role in anti-tumor immunity. Our study showed that XCL1+ CD8+ T cells and their related population are candidates targeting populations to develop promising immunotherapy to improve the clinical outcome of HCC patients.

## Data Availability

The raw data used in this study is public, and the processed data can be made available on request by contacting the corresponding author.
